# Some chalcones derived from thio­phene-3-carbaldehyde: synthesis and crystal structures

**DOI:** 10.1107/S2056989019007503

**Published:** 2019-06-04

**Authors:** Trung Vu Quoc, Duong Tran Thi Thuy, Thuan Dang Thanh, Thanh Phung Ngoc, Vuong Nguyen Thien, Chinh Nguyen Thuy, Luc Van Meervelt

**Affiliations:** a Faculty of Chemistry, Hanoi National University of Education, 136 Xuan Thuy, Cau Giay, Hanoi, Vietnam; b Bien Hoa Gifted High School, 86 Chu Van An Street, Phu Ly City, Ha Nam Province, Vietnam; c C Hai Hau High School, Con Town, Hai Hau District, Nam Dinh Province, Vietnam; d Institute for Tropical Technology, Vietnam Academy of Science and Technology, 18 Hoang Quoc Viet, Cau Giay, Hanoi, Vietnam; e Graduate University of Science and Technology, VAST, 18 Hoang Quoc Viet, Cau Giay, Hanoi, Vietnam; f Department of Chemistry, KU Leuven, Biomolecular Architecture, Celestijnenlaan 200F, Leuven (Heverlee), B-3001, Belgium

**Keywords:** crystal structure, *α,β*-unsaturated carbon­yl, chalcone, thio­phene, Hirshfeld analysis

## Abstract

The synthesis, spectroscopic data and crystal and mol­ecular structures of four 3-(3-phenyl­prop-1-ene-3-one-1-yl)thio­phene derivatives, namely 1-(4-hydroxy­phen­yl)-3-(thio­phen-3-yl)prop-1-en-3-one, C_13_H_10_O_2_S, (**1**), 1-(4-meth­oxy­phen­yl)-3-(thio­phen-3-yl)prop-1-en-3-one, C_14_H_12_O_2_S, (**2**), 1-(4-eth­oxy­phen­yl)-3-(thio­phen-3-yl)prop-1-en-3-one, C_15_H_14_O_2_S, (**3**), and 1-(4-­bromophen­yl)-3-(thio­phen-3-yl)prop-1-en-3-one, C_13_H_9_BrOS, (**4**), are described. The four chalcones have been synthesized by reaction of thio­phene-3-carbaldehyde with an aceto­phenone derivative in an absolute ethanol solution containing potassium hydroxide, and differ in the substituent at the *para* position of the phenyl ring: –OH for **1**, –OCH_3_ for **2**, –OCH_2_CH_3_ for **3** and –Br for **4**. The thio­phene ring in **4** was found to be disordered over two orientations with occupancies 0.702 (4) and 0.298 (4). The configuration about the C=C bond is *E*. The thio­phene and phenyl rings are inclined by 4.73 (12) for **1**, 12.36 (11) for **2**, 17.44 (11) for **3** and 46.1 (6) and 48.6 (6)° for **4**, indicating that the –OH derivative is almost planar and the –Br derivative deviates the most from planarity. However, the substituent has no real influence on the bond distances in the α,β-unsaturated carbonyl moiety. The mol­ecular packing of **1** features chain formation in the *a*-axis direction by O—H⋯O contacts. In the case of **2** and **3**, the packing is characterized by dimer formation through C—H⋯O inter­actions. In addition, C—H⋯π(thio­phene) inter­actions in **2** and C—H⋯S(thio­phene) inter­actions in **3** contribute to the three-dimensional architecture. The presence of C—H⋯π(thio­phene) contacts in the crystal of **4** results in chain formation in the *c*-axis direction. The Hirshfeld surface analysis shows that for all four derivatives, the highest contribution to surface contacts arises from contacts in which H atoms are involved.

## Abstract 2

A series of 3-(3-phenyl­prop-1-ene-3-one-1-yl)thio­phene derivatives containing –OH, –OCH_3_, –OCH_2_CH_3_ and –Br at the *para*-position of the phenyl ring have been synthesized and their mol­ecular and crystal structures are reported.

## Chemical context   

Chalcones, typically referred to as Michael acceptors, can react with nucleophiles at the electrophilic *β*-position of the unsaturated system (Amslinger, 2010 ▸). Many chalcone deriv­atives containing an *α,β*-unsaturated carbonyl show potential biological applications such as being effective against amyloid *β*-induced cytotoxicity (Bukhari *et al.*, 2014 ▸) and irreversibly angiotensin-converting enzyme inhibitors (Hea-Young Park Choo *et al.*, 2000 ▸).

Thio­phene, C_4_H_4_S, belongs to a class of aromatic five-membered heterocycles containing one S heteroatom. Many thio­phene derivatives exhibit biological activities: anti­bacterial (Mishra *et al.*, 2012 ▸), anti­allergic (Gillespie *et al.*, 1985 ▸), analgesic (Laddi *et al.*, 1998 ▸), and act as anti-inflammatory agents (Ferreira *et al.*, 2006 ▸), anti­oxidant agents (Jarak *et al.*, 2005 ▸) and anti­tumor agents (Gadad *et al.*, 1994 ▸). With the introduction of a thio­phene ring into chalcones, it was hoped to design chalcones with inter­esting new structures and properties. The addition of the thio­phene ring to an *α,β*-unsaturated carbonyl group has also been investigated for a substitution at the Cα atom of the thio­phene ring (Harrison *et al.*, 2006 ▸).

Recently, some thio­phene derivatives, such as *N*-(4-oxo-2-sulfanyl­idene-1,3-thia­zolidin-3-yl)-2-(thio­phen-3-yl)acetamide (Vu Quoc *et al.*, 2017 ▸) and 4-phenyl-3-(thio­phen-3-yl-meth­yl)-1*H*-1,2,4-triazole-5(4*H*)-thione (Vu Quoc *et al.*, 2018 ▸), were synthesized by us and their crystal structures were investigated by X-ray diffraction.
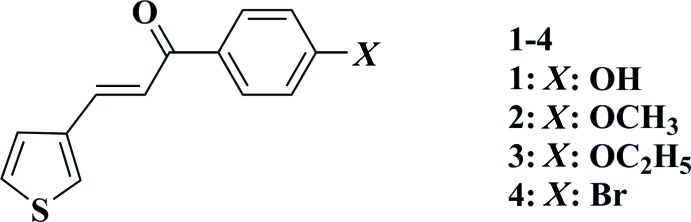


In this study, we present the synthesis and crystal structure of four chalcones (**1**–**4**) containing a thio­phene ring: 3-(3-phenyl­prop-1-ene-3-one-1-yl)thio­phene derivatives contain­ing –OH, –OCH_3_, –OCH_2_CH_3_ and –Br at the *para* position of the phenyl ring.

## Structural commentary   

**Figure 1:**
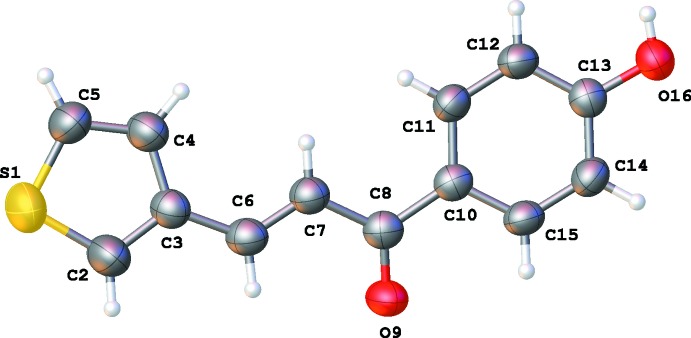
The mol­eculare structure of **1** showing 50% displacement ellipsoids.

The asymmetric units of **1**, **2**, **3** and **4** are illustrated in Figs. 1 ▸, 2 ▸, 3 ▸ and 4 ▸, respectively. The thio­phene group in **4** is disordered over two orientations by a rotation of about 180° about the C3—C6 bond in a 0.702 (4): 0.298 (4) ratio. Chalcone **1** bearing the –OH substituent is almost planar, with the dihedral angle between the thio­phene and phenyl rings being 4.73 (12)°. For the other chalcones, the deviation from planarity is significant, as illustrated by the dihedral angles: 12.36 (11)° for **2**, 17.44 (11)° for **3** and 46.1 (6) and 48.6 (6)° for **4**. The C6=C7 bond lengths [1.329 (3) Å for **1**, 1.328 (3) Å for **2**, 1.319 (3) Å for **3** and 1.325 (5) Å for **4**] are almost identical. The configuration of the C6=C7 bond can be described as *E* [torsion angles C3—C6—C7—C8 are −175.4 (2), −177.8 (2), 179.75 (18) and −174.3 (3)° for **1**–**4**, respectively]. For **1**, this *E* configuration gives rise to an intra­molecular C6—H6⋯O9 inter­action (Table 1 ▸). The substituent at the *para*-position of the phenyl ring has no significant influence on the C8=O9 bond length [1.232 (3) Å in **1**, 1.228 (3) Å in **2**, 1.224 (2) Å in **3** and 1.224 (4) Å in **4**].

## Supra­molecular features   

**Figure 5:**
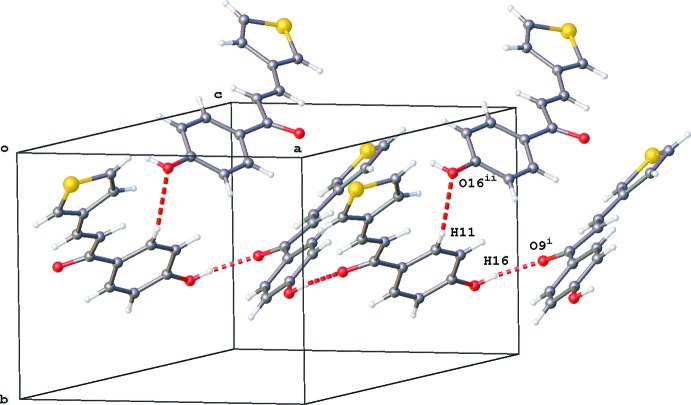
Partial crystal packing of **1** showing the inter­molecular hydrogen-bonding inter­actions as red dashed lines (see Table 1 ▸ for details).

In chalcone derivative **1**, which crystallizes in the ortho­rhom­bic space group *Pbca*, the –OH substituent is involved as donor in inter­molecular O16—H16⋯O9^i^ [symmetry code: (i) *x* + 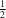, *y*, 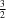 − *z*] hydrogen bonding, resulting in the formation of chains of mol­ecules running in the *a*-axis direction (Fig. 5 ▸, Table 1 ▸). As acceptor, the –OH substituent inter­acts by inter­molecular C11—H11⋯O16 hydrogen bonding (Fig. 5 ▸, Table 1 ▸).

**Figure 6:**
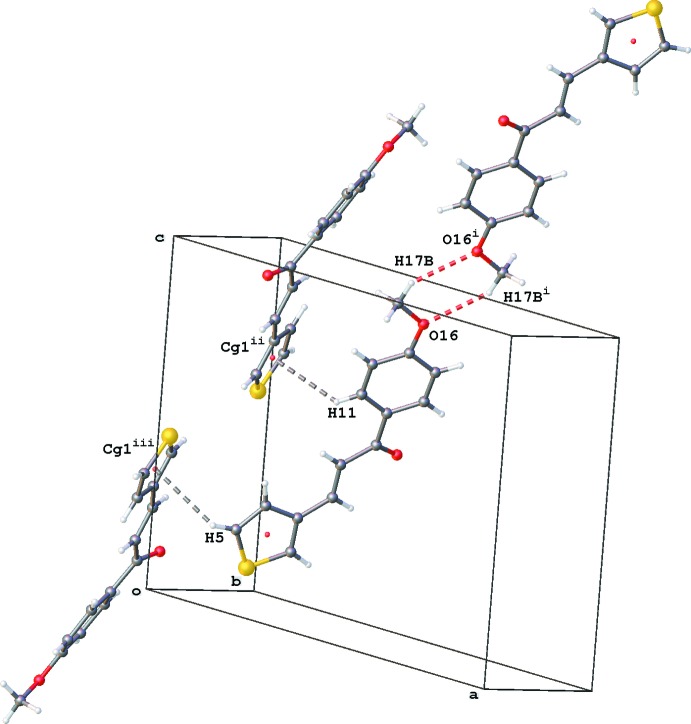
Partial crystal packing of **2** showing dimer formation through C—H⋯O (red dashed lines) and C—H⋯π inter­actions [grey dashed lines; *Cg*1 is the centroid of the thio­phene ring; symmetry codes: (i) −*x* + 1, −*y* + 2, *z* + 2, (ii) *x*, −*y* + 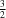, *z* + 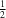, (iii) −*x*, *y* − 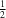, −*z* + 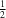].

Crystals of **2**–**4** belong to the monoclinic space group *P*2_1_/*c*. The crystal packing of **2** is characterized by inversion-dimer formation between the meth­oxy groups by weak C17—H17*B*⋯O16^i^ inter­actions [H17⋯O16^i^ = 2.61 Å; symmetry code (i): −*x* + 1, −*y* + 2, −*z* + 2] and C—H⋯π(thio­phene) inter­actions (C5—H5⋯*Cg*1^ii^ and C11—H11⋯*Cg*1^iii^; for details see Table 2 ▸ and Fig. 6 ▸).

**Figure 7:**
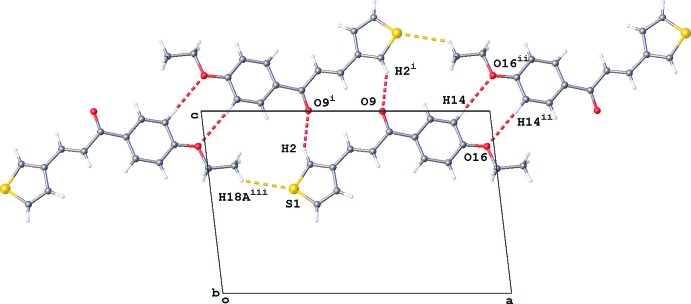
Formation of sheets of mol­ecules of **3** by C—H⋯O and C—H⋯S inter­actions [red dashed lines; symmetry codes: (i) −*x* + 1, −*y* + 1, −*z* + 2, (ii) −*x* + 2, −*y* + 1, −*z* + 2, (iii) *x* − 1, *y*, *z*]

In the packing of **3**, C2—H2⋯O9^i^ inter­actions result in dimeric units forming rings of 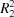
(14) graph-set motif [symmetry code (i): 1 − *x*, 1 − *y*, 2 − *z*; Table 3 ▸, Fig. 7 ▸]. In addition, two weaker inter­actions are present in the packing. Inversion dimers are formed by C14—H14⋯O16^ii^ inter­actions [H14⋯O16^ii^ = 2.71 Å; symmetry code: (ii) −*x* + 2, −*y* + 1, −*z* + 2] enclosing an 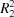
(8) ring motif. Chains of mol­ecules running in the *a*-axis direction are the consequence of C18^iii^—H18*A* ^iii^⋯S1 inter­actions [H18*A* ^iii^⋯S1 = 3.05 Å; symmetry code: (iii) *x* − 1, *y*, *z*]. These inter­molecular inter­actions result in the formation of sheets of mol­ecules parallel to the *ac* plane (Fig. 7 ▸).

**Figure 8:**
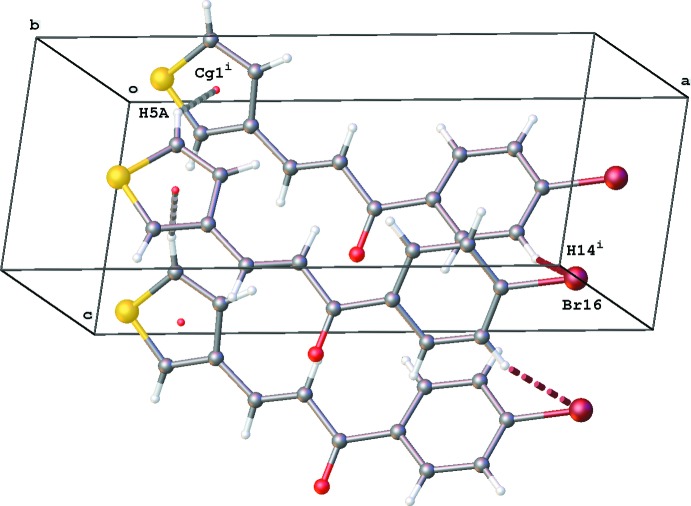
Chains of mol­ecules in **4** running in the *c*-axis direction formed by C5*A*—H5*A*⋯*Cg*1^i^ inter­actions [grey dashed lines, *Cg*1 is the centroid of the major-disorder component of the thio­phene ring; symmetry code: (i) *x*, 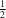 − *y*, *z* − 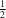].

In the packing of **4**, chains running in the *c*-axis direction are formed by C5—H5⋯π(thio­phene) inter­actions (Table 4 ▸, Fig. 8 ▸). At the other side of the mol­ecule, the closest contact for the Br16 atom is with H14 [Br16⋯H14^i^ = 3.23 Å; Fig.8]. The shortest Br⋯Br distance [4.4621 (11) Å] in the crystal packing is Br16⋯Br16^ii^ [symmetry code: (ii) −*x* + 2, −*y* + 1, −*z* + 2].

No voids or π–π stackings are observed in the crystal packing of **1**–**4**.

## Database survey   

A search of the Cambridge Structural Database (CSD, Version 5.40, update of February 2019; Groom *et al.*, 2016 ▸) for 3-(3-thien­yl)prop-2-en-1-one gave three hits, *viz*. AYUPIU (Shalini *et al.*, 2011 ▸), IBIRUJ (Oyarce *et al.*, 2017 ▸) and UNAJIE (Baggio *et al.*, 2016 ▸).

The configuration about the double bonds in the symmet­rical 1,5-bis­(thio­phen-3-yl)penta-1,4-dien-3-one (AYUPIU; Shalini *et al.*, 2011 ▸) is twice *E*. The dihedral angle between the terminal thio­phene rings is 15.45 (10)°. In the crystal packing, C—H⋯O inter­actions link the mol­ecules into arrays in the *ac* plane that are further connected by C—H⋯π inter­actions.

Both thio­phene rings in 3-hy­droxy-1-(thio­phen-2-yl)-3-(thio­phen-3-yl)prop-2-en-1-one (IBIRUJ; Oyarce *et al.*, 2017 ▸) are disordered; the major-disorder components are inclined to each other by 12.1 (3)°. Chains of mol­ecules running in the *c*-axis direction are formed through C—H⋯O inter­actions.

In the crystal of 1,3-bis­(3-thien­yl)prop-2-en-1-one (UNAJIE; Baggio *et al.*, 2016 ▸), the stereochemistry about the double bond is *E* and the dihedral angle between the thio­phene rings is 8.88 (10)°. Columns of stacking mol­ecules along [010] indicate that π–π inter­actions play an important role in the crystal packing, together with C—H⋯O hydrogen bonds between the columns.

A search for 1-phenyl-3-(2-thien­yl)prop-2-en-1-one allowing substitution at the phenyl ring resulted in 19 hits of which the compound 1-(4-bromo­phen­yl)-3-(2-thien­yl)prop-2-en-1-one (GENXED; Patil *et al.*, 2006 ▸; GENXED01; Arshad *et al.*, 2017 ▸) is the 2-thienyl derivative of **4**. In addition to similar cell parameters, the thio­phene ring also shows rotational disorder [ratio 0.791 (2):0.209 (2) for GENXED; Patil *et al.*, 2006 ▸] and the angles between thio­phene and phenyl rings are comparable [46.49 (11) and 48.4 (3)° for GENXED; Patil *et al.*, 2006 ▸].

## Hirshfeld surface analysis   

**Figure 9:**
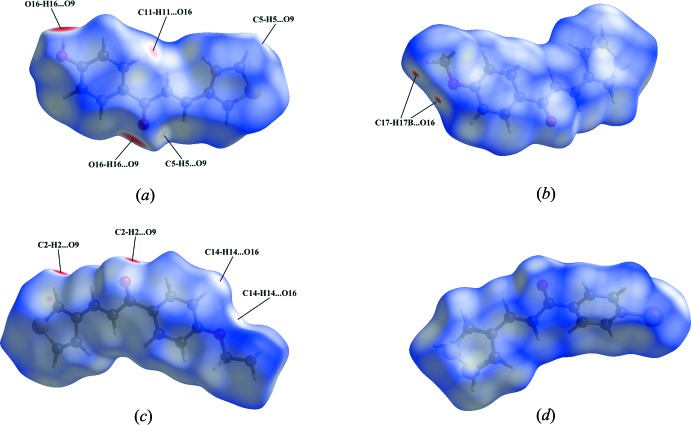
The Hirshfeld surface mapped over *d*_norm_ for (*a*) compound **1** in the range −0.704 to 1.267 a.u., (*b*) compound **2** in the range −0.059 to 1.101 a.u., (*c*) compound **3** in the range −0.200 to 1.439 a.u. and (*d*) compound **4** in the range 0.007 to 0.942 a.u.

The Hirshfeld surface analysis (Spackman & Jayatilaka, 2009 ▸) and the associated two-dimensional fingerprint plots (McKinnon *et al.*, 2007 ▸) were performed using *CrystalExplorer* (Turner *et al.*, 2017 ▸). The Hirshfeld surfaces of compounds **1**–**4** mapped over *d*_norm_ are given in Fig. 9 ▸. The relative distributions from the different inter­atomic contacts to the Hirshfeld surfaces are presented in Table 5 ▸.

The bright-red spots in Fig. 9 ▸ *a* near atoms O16 and O9 are indicative for the O16—H16⋯O9 hydrogen bond in the crystal packing of **1**. The additional faint-red spots illustrate C—H⋯O inter­actions. The most significant contributions to the Hirshfeld surface are from C⋯H/H⋯C (30.4%), H⋯H (28.8%) and O⋯H/H⋯O (18.5%) contacts (Table 5 ▸).

For compound **2**, the donor and acceptor of the relatively weak C17—H17*B*⋯O16 inter­action are viewed as diminutive red spots near atoms H17*B* and O16 in Fig. 9 ▸ *b*. The C—H⋯π(thio­phene) inter­actions are indicated by the high contribution from C⋯H/H⋯C contacts (33.9%) to the Hirshfeld surface (Table 5 ▸).

The bright-red spots in Fig. 9 ▸ *c* near atoms O9 and H2 of **3** refer to the strong C2—H2⋯O9 dimer formation, while the faint-red spots near atoms O16 and H14 are indicative for the relatively weak C14—H14⋯O16 dimer formation. Near atom C2 another faint-red spot refers to a contact (2.73 Å) with atom H5.

The Hirshfeld surface mapped over *d*_norm_ for **4** (Fig. 9 ▸ *d*) shows no short inter­atomic contacts. Again the C—H⋯π inter­action with the disordered thio­phene ring is reflected in the high contribution from C⋯H/H⋯C contacts (32.6%) to the Hirshfeld surface (Table 5 ▸).

For the four derivatives, the largest contributions of inter­atomic contacts to the Hirshfeld surface are contacts in which H atoms are involved (Table 5 ▸).

## Synthesis and crystallization   

**Figure 10:**
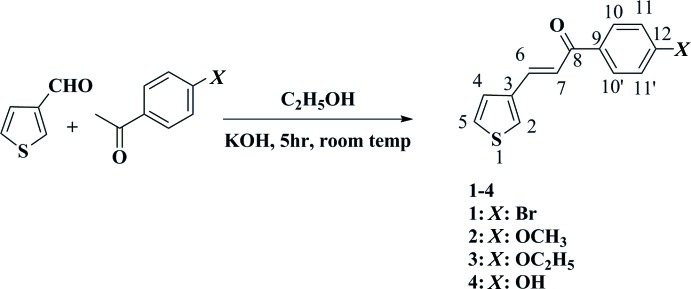
Reaction scheme for the title compounds **1**–**4**.

The reaction scheme to synthesize the title compounds **1**–**4** is given in Fig. 10 ▸.

***Synthesis of α,β-unsaturated ketone compounds 1**–**4:***

In a 250 mL beaker, thio­phene-3-carbaldehyde (0.1 mole) and substituted aceto­phenone (0.1 mol) were dissolved in ethanol (100 mL). To this mixture, a 50% KOH (10 mL) solution was added and the mixture was stirred by a magnetic stirrer for 5 h at room temperature until a precipitate appeared. The products **1**–**4** were obtained as solids, which were filtered under low pressure and recrystallized from ethanol.

***Data for 3-(3-(4-hy­droxy­phen­yl)prop-1-ene-3-one-1-yl)thio­phene (1):***

Yellow crystals; yield 90%; m.p. 388 K; IR (Nicolet Impact 410 FT–IR, KBr, cm^−1^): 3456.8 (OH), 2983.3 (CH aromatic, alkene), 1643.1 (C=O), 1596.8 (C=C, C=N), 1037.4 [–CH=(*trans*)]; ^1^H NMR [Bruker XL-500, 500 MHz, *d*_6_-CDCl_3_, δ (ppm), *J* (Hz)]: 6.93 (*d*, 2H, *J* = 9.0, H11,11′), 7.34 (*d*, 1H, *J* = 15.5, H7), 7.37 (*d*, 1H, *J* = 2, *J* = 3, H2), 7.41 (*dd*, 1H, *J* = 5, H4), 7.59 (*dd*, 1H, *J* = 5.5, H5), 7.79 (*d*, 1H, *J* = 15.5, H6), 7.98 (*d*, 2H, *J* = 8.5, H10,10′). ^13^C NMR [Bruker XL-500, 125 MHz, *d*_6_-CDCl_3_, δ (ppm)]: 121.63 (C2); 128.79 (C3), 126.98 (C4); 125.26 (C5); 131.39 (C6); 131.07 (C7); 189.0 (C8); 159.84 (C9); 138.33 (C10,10′); 137.64 (C11,11′); 115.4 (C12). Calculation for C_13_H_10_O_2_S: *M* = 230 au.

***Data for 3-(3-(4-meth­oxy­phen­yl)prop-1-ene-3-one-1-yl)thio­phene (2):***

White crystals; yield 70%; m.p. 378 K; IR (Nicolet Impact 410 FT–IR, KBr, cm^−1^): 3009.3 (CH alkane), 2974.3 (CH aromatic, alkene), 1651.1 (C=O), 1597.5 (C=C, C=N), 1017.2 [–CH=(*trans*)]; ^1^H NMR [Bruker XL-500, 500 MHz, *d*_6_-CDCl_3_, δ (ppm), *J* (Hz)]: 3.89 (*s*, 3H, OCH_3_), 6.98 (*d*, 2H, *J* = 9.0, H11,11′), 7.35 (*d*, 1H, *J* = 15.5, H7), 7.36 (*dd*, 1H, *J* = 2.5, *J* = 5, H2), 7.42 (*d*, 1H, *J* = 5, H4), 7.58 (*dd*, 1H, *J* = 2.5, H5), 7.79 (*d*, 1H, *J* = 16, H6), 8.02 (*d*, 2H, *J* = 9, H10,10′). ^13^C NMR [Bruker XL-500, 125 MHz, *d*_6_-CDCl_3_, δ (ppm)]: 121.70 (C2), 128.68 (C3), 126.94 (C4), 125.28 (C5), 131.19 (C6), 130.74 (C7), 188.96 (C8), 163.41 (C9), 138.37 (C10,10′), 137.44 (C11,11′), 113.85 (C12), 55.5 (OCH_3_). Calculation for C_14_H_12_O_2_S: *M* = 244 au.

***Data for 3-(3-(4-eth­oxy­phen­yl)prop-1-ene-3-one-1-yl)thio­phene (3):***

White crystals; yield 50%; m.p. 380 K; IR (Nicolet Impact 410 FT–IR, KBr, cm^−1^): 3010.6 (CH alkane), 2983.3 (CH aromatic, alkene), 1657.1 (C=O), 1596.7 (C=C, C=N), 1011.4 [–CH=(*trans*)]; ^1^H NMR [Bruker XL-500, 500 MHz, *d*_6_-CDCl_3_, δ (ppm), *J* (Hz)]: 1.53 (*t*, 3H, *J* = 7, OCH_2_C*H*_3_), 4.12 (*q*, 2H, *J* = 7, 7, OC*H*_2_CH_3_), 6.96 (*d*, 2H, *J* = 9.0, H11,11′), 7.36 (*d*, 1H, *J* = 15.5, H7), 7.36 (*d*, 1H, *J* = 2, *J* = 3, H2), 7.42 (*dd*, 1H, *J* = 1.5, *J* = 5, H4), 7.58 (*dd*, 1H, *J* = 1.5, *J* = 5.5, H5), 7.78 (*d*, 1H, *J* = 15.5, H6), 8.01 (*d*, 2H, *J* = 9, H10,10′). ^13^C NMR [Bruker XL-500, 125 MHz, *d*_6_-CDCl_3_, δ (ppm)]: 121.73 (C2); 128.63 (C3), 126.93 (C4); 125.29 (C5); 131.00 (C6); 130.75 (C7); 188.96 (C8); 162.85 (C9); 138.4 (C10,10′); 137.37 (C11,11′); 114.3 (C12); 63.80 (O*C*H_2_CH_3_); 14.7 (OCH_2_ *C*H_3_). Calculation for C_15_H_14_O_2_S: *M* = 258 au.

***Data for 3-(3-(4-bromo­phen­yl)prop-1-ene-3-one-1-yl)thio­phene (4):***

Bright-yellow crystals; yield 99%; m.p. 353 K; IR (Nicolet Impact 410 FT–IR, KBr, cm^−1^): 3090.7 (CH aromatic, alkene), 1654.5 (C=O), 1595.8 (C=C, C=N), 1006.1 [–CH=(*trans*)]; ^1^H NMR [Bruker XL-500, 500 MHz, *d*_6_-CDCl_3_, δ (ppm), *J* (Hz)]: 6.69 (*d*, 2H, *J* = 9.0, H11,11′), 7.35 (*d*, 1H, *J* = 15.5, H7), 7.36 (*dd*, 1H, *J* = 3, *J* = 5.5, H2), 7.41 (*d*, 1H, *J* = 5.5, H4), 7.56 (*dd*, 1H, *J* = 3, H5), 7.77(*d*, 1H, *J* = 15.5, H6), 7.91 (*d*, 2H, *J* = 8.5, H10,10′). ^13^C NMR [Bruker XL-500, 125 MHz, *d*_6_-CDCl_3_, δ (ppm)]: 121.29 (C2), 127.81 (C3), 127.18 (C4), 125.21 (C5), 129.97 (C6), 131.93 (C7), 189.66 (C8), 138.81 (C9), 138.81 (C10,10′), 138.05(C11,11′), 129.50 (C12). Calculation for C_13_H_9_OSBr: *M* = 293 au.

## Refinement   

Crystal data, data collection and structure refinement details are summarized in Table 6 ▸.

All H atoms were placed in idealized positions and refined in riding mode, with *U*_iso_(H) values assigned as 1.2*U*_eq_ of the parent atoms (1.5 times for methyl groups), with C—H distances of 0.93 (aromatic and =CH), 0.96 (CH_3_) and 0.97 Å (CH_2_), and O—H distances of 0.82 Å (rotating OH).

In **4**, the thio­phene ring was disordered over two positions [population parameters 0.702 (4) and 0.298 (4)] and was refined with restraints for the bond lengths and angles in the ring. The anisotropic temperature factors for atoms S1, C2, C4 and C5 in both orientations were constrained to be equal. In the final cycles of refinement, two and one outliers were omitted for **1** and **2**, respectively.

## Supplementary Material

Crystal structure: contains datablock(s) 1, 2, 3, 4. DOI: 10.1107/S2056989019007503/lh5905sup1.cif

Structure factors: contains datablock(s) 1. DOI: 10.1107/S2056989019007503/lh59051sup2.hkl

Structure factors: contains datablock(s) 2. DOI: 10.1107/S2056989019007503/lh59052sup3.hkl

Structure factors: contains datablock(s) 3. DOI: 10.1107/S2056989019007503/lh59053sup4.hkl

Structure factors: contains datablock(s) 4. DOI: 10.1107/S2056989019007503/lh59054sup5.hkl

Supporting information file. DOI: 10.1107/S2056989019007503/lh59051sup6.cml

Supporting information file. DOI: 10.1107/S2056989019007503/lh59052sup7.cml

Supporting information file. DOI: 10.1107/S2056989019007503/lh59053sup8.cml

Supporting information file. DOI: 10.1107/S2056989019007503/lh59054sup9.cml

CCDC references: 1918095, 1918094, 1918093, 1918092

Additional supporting information:  crystallographic information; 3D view; checkCIF report

## Figures and Tables

**Figure 2:**
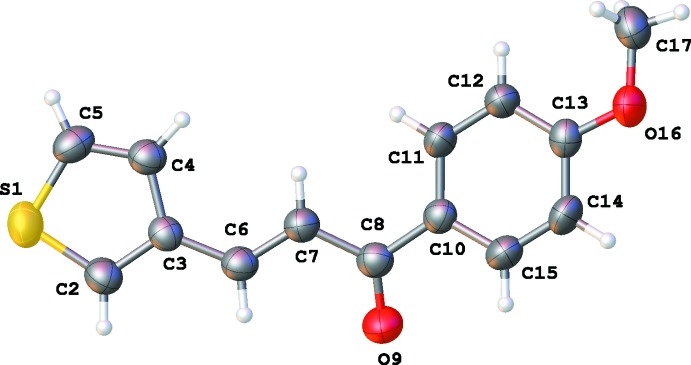
The mol­eculare structure of **2** showing 50% displacement ellipsoids.

**Figure 3:**
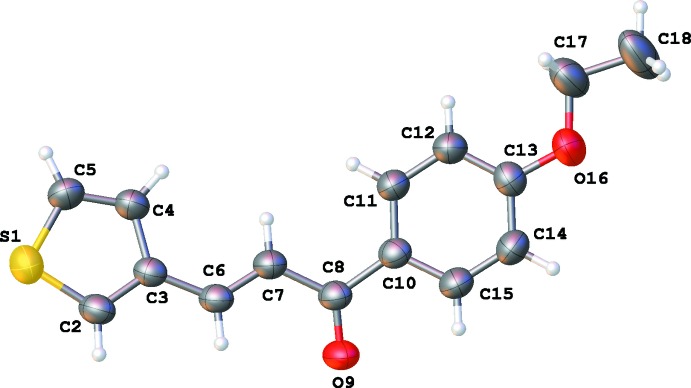
The mol­eculare structure of **3** showing 50% displacement ellipsoids.

**Figure 4:**
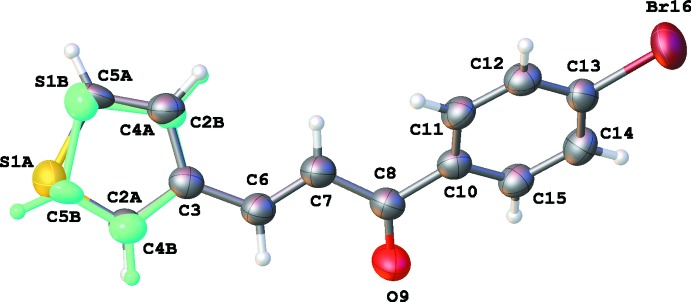
The mol­eculare structure of **4** showing 50% displacement ellipsoids. The minor-disorder component is shown in light blue.

**Table 1:** Hydrogen-bond geometry (Å, °) for **1** [Chem scheme1]

*D*—H⋯*A*	*D*—H	H⋯*A*	*D*⋯*A*	*D*—H⋯*A*
O16—H16⋯O9^i^	0.82	1.86	2.667 (2)	167
C6—H6⋯O9	0.93	2.46	2.785 (3)	100
C11—H11⋯O16^ii^	0.93	2.55	3.425 (3)	157

Symmetry codes: (i) 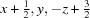; (ii) 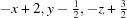.

**Table 2:** Hydrogen-bond geometry (Å, °) for **2** [Chem scheme1] *Cg*1 is the centroid of the S1/C2–C5 ring.

*D*—H⋯*A*	*D*—H	H⋯*A*	*D*⋯*A*	*D*—H⋯*A*
C5—H5⋯*Cg*1^ii^	0.93	2.94	3.602 (2)	129
C11—H11⋯*Cg*1^iii^	0.93	2.99	3.598 (2)	125

Symmetry codes: (ii) 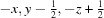; (iii) 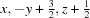.

**Table 3:** Hydrogen-bond geometry (Å, °) for **3** [Chem scheme1]

*D*—H⋯*A*	*D*—H	H⋯*A*	*D*⋯*A*	*D*—H⋯*A*
C2—H2⋯O9^i^	0.93	2.47	3.324 (2)	153

Symmetry code: (i) 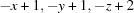.

**Table 4:** Hydrogen-bond geometry (Å, °) for **4** [Chem scheme1] *Cg*1 and *Cg*2 are the centroids of the major- and minor-disorder components of the thio­phene ring, respectively.

*D*—H⋯*A*	*D*—H	H⋯*A*	*D*⋯*A*	*D*—H⋯*A*
C5*A*—H5*A*⋯*Cg*1^i^	0.93	2.80	3.493 (14)	132
C5*A*—H5*A*⋯*Cg*2^i^	0.93	2.85	3.52 (2)	130

Symmetry code: (i) 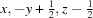.

**Table 5:** Percentage contributions of inter­atomic contacts to the Hirshfeld surfaces for compounds (**1**–**4**)

Contact	**1**	**2**	**3**	**4**
H⋯H	28.8	33.5	44.5	28.5
S⋯H/H⋯S	13.0	11.6	10.2	3.9
C⋯H/H⋯C	30.4	33.9	22.1	32.6
O⋯H/H⋯O	18.5	15.8	13.8	11.1
C⋯C	3.2	0.7	4.5	2.4
C⋯S/S⋯C	3.7	0.9	3.5	0.0
S⋯S	0.0	1.5	0.0	0.0
S⋯O/O⋯S	0.3	0.0	0.1	0.0
C⋯O/O⋯C	1.8	1.6	1.0	1.0
O⋯O	0.0	0.6	0.0	0.0
Br⋯S/S⋯Br				2.8
Br⋯C/C⋯Br				0.6
Br⋯H/H⋯Br				16.5
Br⋯O/O⋯Br				0.0
Br⋯Br				0.5

**Table 6:** Experimental details

	**1**	**3**	**2**	**4**
Crystal data				
Chemical formula	C_13_H_10_O_2_S	C_14_H_12_O_2_S	C_15_H_14_O_2_S	C_13_H_9_BrOS
*M*_r_	230.27	244.30	258.32	293.17
Crystal system, space group	Orthorhombic, *P* *b* *c* *a*	Monoclinic, *P*2_1_/*c*	Monoclinic, *P*2_1_/*c*	Monoclinic, *P*2_1_/*c*
Temperature (K)	294	294	294	293
*a*, *b*, *c* (Å)	11.0808 (5), 9.0251 (5), 22.8157 (10)	16.4118 (13), 5.8387 (5), 12.6456 (9)	16.5120 (8), 7.7851 (5), 10.4913 (5)	14.1245 (7), 14.2016 (13), 5.8809 (4)
α, β, γ (°)	90, 90, 90	90, 97.279 (7), 90	90, 96.813 (4), 90	90, 98.081 (6), 90
*V* (Å^3^)	2281.69 (19)	1201.98 (16)	1339.11 (13)	1167.93 (15)
*Z*	8	4	4	4
Radiation type	Mo *K*α	Mo *K*α	Mo *K*α	Mo *K*α
μ (mm^−1^)	0.26	0.26	0.23	3.67
Crystal size (mm)	0.4 × 0.3 × 0.07	0.45 × 0.3 × 0.15	0.5 × 0.35 × 0.15	0.4 × 0.4 × 0.05

Data collection				
Diffractometer	SuperNova, single source at offset/far, Eos	SuperNova, single source at offset/far, Eos	SuperNova, single source at offset/far, Eos	SuperNova, single source at offset/far, Eos
Absorption correction	Multi-scan (*CrysAlis PRO*; Rigaku OD, 2018 ▸)	Multi-scan (*CrysAlis PRO*; Rigaku OD, 2018 ▸)	Multi-scan (*CrysAlis PRO*; Rigaku OD, 2018 ▸)	Multi-scan (*CrysAlis PRO*; Rigaku OD, 2018 ▸)
*T*_min_, *T*_max_	0.522, 1.000	0.803, 1.000	0.733, 1.000	0.367, 1.000
No. of measured, independent and observed [*I* > 2σ(*I*)] reflections	9745, 2333, 1814	5075, 2457, 1771	13246, 2734, 2162	12050, 2392, 1683
*R*_int_	0.019	0.021	0.035	0.045
(sin θ/λ)_max_ (Å^−1^)	0.625	0.625	0.625	0.625

Refinement				
*R*[*F* ^2^ > 2σ(*F* ^2^)], *wR*(*F* ^2^), *S*	0.049, 0.134, 1.05	0.046, 0.118, 1.04	0.047, 0.141, 1.05	0.043, 0.107, 1.02
No. of reflections	2333	2457	2734	2392
No. of parameters	146	156	165	158
No. of restraints	0	0	0	20
H-atom treatment	H-atom parameters constrained	H-atom parameters constrained	H-atom parameters constrained	H-atom parameters constrained
Δρ_max_, Δρ_min_ (e Å^−3^)	0.20, −0.35	0.16, −0.26	0.19, −0.28	0.40, −0.46

Computer programs: *CrysAlis PRO* (Rigaku OD, 2018 ▸), *SHELXT* (Sheldrick, 2015*a* ▸), *SHELXL* (Sheldrick, 2015*b* ▸) and *OLEX2* (Dolomanov *et al.*, 2009 ▸).

## supplementary crystallographic information

### 1-(4-Hydroxyphenyl)-3-(thiophen-3-yl)prop-1-en-3-one (1) Crystal data

**Table:**

C_13_H_10_O_2_S	*D*_x_ = 1.341 Mg m^−^^3^
*M**_r_* = 230.27	Mo *K*α radiation, λ = 0.71073 Å
Orthorhombic, *P**b**c**a*	Cell parameters from 3900 reflections
*a* = 11.0808 (5) Å	θ = 3.0–27.0°
*b* = 9.0251 (5) Å	µ = 0.26 mm^−^^1^
*c* = 22.8157 (10) Å	*T* = 294 K
*V* = 2281.69 (19) Å^3^	Block, yellow
*Z* = 8	0.4 × 0.3 × 0.07 mm
*F*(000) = 960	

### 1-(4-Hydroxyphenyl)-3-(thiophen-3-yl)prop-1-en-3-one (1) Data collection

**Table:**

SuperNova, single source at offset/far, Eos diffractometer	2333 independent reflections
Radiation source: micro-focus sealed X-ray tube, SuperNova (Mo) X-ray Source	1814 reflections with *I* > 2σ(*I*)
Mirror monochromator	*R*_int_ = 0.019
Detector resolution: 15.9631 pixels mm^-1^	θ_max_ = 26.4°, θ_min_ = 2.6°
ω scans	*h* = −13→13
Absorption correction: multi-scan (CrysAlisPro; Rigaku OD, 2018)	*k* = −4→11
*T*_min_ = 0.522, *T*_max_ = 1.000	*l* = −27→28
9745 measured reflections	

### 1-(4-Hydroxyphenyl)-3-(thiophen-3-yl)prop-1-en-3-one (1) Refinement

**Table:**

Refinement on *F*^2^	0 restraints
Least-squares matrix: full	Hydrogen site location: inferred from neighbouring sites
*R*[*F*^2^ > 2σ(*F*^2^)] = 0.049	H-atom parameters constrained
*wR*(*F*^2^) = 0.134	*w* = 1/[σ^2^(*F*_o_^2^) + (0.0463*P*)^2^ + 1.4972*P*] where *P* = (*F*_o_^2^ + 2*F*_c_^2^)/3
*S* = 1.05	(Δ/σ)_max_ < 0.001
2333 reflections	Δρ_max_ = 0.20 e Å^−^^3^
146 parameters	Δρ_min_ = −0.35 e Å^−^^3^

### 1-(4-Hydroxyphenyl)-3-(thiophen-3-yl)prop-1-en-3-one (1) Special details

**Table:**

Geometry. All esds (except the esd in the dihedral angle between two l.s. planes) are estimated using the full covariance matrix. The cell esds are taken into account individually in the estimation of esds in distances, angles and torsion angles; correlations between esds in cell parameters are only used when they are defined by crystal symmetry. An approximate (isotropic) treatment of cell esds is used for estimating esds involving l.s. planes.

### 1-(4-Hydroxyphenyl)-3-(thiophen-3-yl)prop-1-en-3-one (1) Fractional atomic coordinates and isotropic or equivalent isotropic displacement parameters (Å^2^)

**Table:**

	*x*	*y*	*z*	*U*_iso_*/*U*_eq_	
S1	0.89495 (8)	0.20994 (11)	0.39007 (3)	0.0907 (3)	
C2	0.8168 (2)	0.3325 (4)	0.43118 (11)	0.0783 (8)	
H2	0.749344	0.383495	0.417667	0.094*	
C3	0.8629 (2)	0.3490 (3)	0.48637 (9)	0.0533 (6)	
C4	0.9650 (2)	0.2575 (3)	0.49383 (11)	0.0637 (7)	
H4	1.008933	0.252750	0.528507	0.076*	
C5	0.9932 (2)	0.1760 (3)	0.44462 (11)	0.0704 (7)	
H5	1.058032	0.110896	0.441931	0.085*	
C6	0.8079 (2)	0.4425 (3)	0.53031 (10)	0.0550 (6)	
H6	0.738492	0.493292	0.519204	0.066*	
C7	0.8460 (2)	0.4637 (3)	0.58489 (9)	0.0521 (5)	
H7	0.918191	0.420470	0.596709	0.062*	
C8	0.77790 (19)	0.5528 (2)	0.62690 (9)	0.0486 (5)	
O9	0.67534 (14)	0.5953 (2)	0.61457 (7)	0.0621 (5)	
C10	0.83162 (18)	0.5889 (2)	0.68496 (9)	0.0458 (5)	
C11	0.9339 (2)	0.5188 (3)	0.70693 (10)	0.0511 (5)	
H11	0.973899	0.449440	0.683877	0.061*	
C12	0.9773 (2)	0.5503 (3)	0.76226 (10)	0.0536 (6)	
H12	1.044085	0.499832	0.776762	0.064*	
C13	0.92110 (19)	0.6570 (2)	0.79603 (9)	0.0477 (5)	
C14	0.8212 (2)	0.7312 (3)	0.77412 (10)	0.0545 (6)	
H14	0.784258	0.804822	0.796302	0.065*	
C15	0.77688 (19)	0.6960 (3)	0.71980 (10)	0.0518 (5)	
H15	0.708722	0.744893	0.705920	0.062*	
O16	0.95988 (15)	0.6936 (2)	0.85077 (6)	0.0601 (5)	
H16	1.025310	0.654151	0.857006	0.090*	

### 1-(4-Hydroxyphenyl)-3-(thiophen-3-yl)prop-1-en-3-one (1) Atomic displacement parameters (Å^2^)

**Table:**

	*U*^11^	*U*^22^	*U*^33^	*U*^12^	*U*^13^	*U*^23^
S1	0.0964 (6)	0.1162 (8)	0.0596 (4)	0.0193 (5)	−0.0060 (4)	−0.0214 (4)
C2	0.0726 (17)	0.104 (2)	0.0583 (15)	0.0212 (17)	−0.0110 (13)	−0.0091 (15)
C3	0.0502 (12)	0.0622 (14)	0.0475 (12)	0.0007 (11)	0.0014 (10)	0.0023 (11)
C4	0.0598 (14)	0.0797 (17)	0.0515 (13)	0.0102 (13)	−0.0034 (11)	0.0004 (12)
C5	0.0670 (15)	0.0795 (19)	0.0647 (16)	0.0183 (15)	0.0016 (13)	−0.0065 (14)
C6	0.0479 (12)	0.0638 (15)	0.0533 (13)	0.0051 (11)	0.0010 (10)	0.0052 (11)
C7	0.0478 (12)	0.0562 (13)	0.0522 (12)	0.0038 (10)	0.0000 (10)	0.0012 (10)
C8	0.0475 (12)	0.0483 (12)	0.0500 (12)	−0.0017 (10)	0.0024 (9)	0.0060 (10)
O9	0.0497 (9)	0.0816 (12)	0.0550 (9)	0.0138 (9)	−0.0052 (7)	−0.0024 (8)
C10	0.0427 (11)	0.0441 (12)	0.0507 (12)	−0.0017 (9)	0.0037 (9)	0.0038 (9)
C11	0.0498 (12)	0.0477 (12)	0.0558 (13)	0.0051 (10)	−0.0003 (10)	−0.0054 (10)
C12	0.0482 (12)	0.0522 (13)	0.0604 (14)	0.0072 (11)	−0.0063 (10)	−0.0014 (11)
C13	0.0466 (11)	0.0492 (12)	0.0474 (11)	−0.0054 (10)	0.0022 (9)	0.0005 (10)
C14	0.0498 (12)	0.0562 (14)	0.0577 (13)	0.0065 (11)	0.0043 (10)	−0.0082 (11)
C15	0.0442 (11)	0.0523 (13)	0.0590 (13)	0.0079 (10)	−0.0022 (10)	0.0005 (11)
O16	0.0580 (10)	0.0712 (11)	0.0510 (9)	0.0040 (9)	−0.0045 (7)	−0.0082 (8)

### 1-(4-Hydroxyphenyl)-3-(thiophen-3-yl)prop-1-en-3-one (1) Geometric parameters (Å, º)

**Table:**

S1—C2	1.689 (3)	C8—C10	1.488 (3)
S1—C5	1.682 (3)	C10—C11	1.392 (3)
C2—H2	0.9300	C10—C15	1.391 (3)
C2—C3	1.367 (3)	C11—H11	0.9300
C3—C4	1.411 (3)	C11—C12	1.380 (3)
C3—C6	1.446 (3)	C12—H12	0.9300
C4—H4	0.9300	C12—C13	1.382 (3)
C4—C5	1.378 (3)	C13—C14	1.387 (3)
C5—H5	0.9300	C13—O16	1.361 (2)
C6—H6	0.9300	C14—H14	0.9300
C6—C7	1.329 (3)	C14—C15	1.370 (3)
C7—H7	0.9300	C15—H15	0.9300
C7—C8	1.461 (3)	O16—H16	0.8200
C8—O9	1.232 (3)		
			
C5—S1—C2	92.31 (13)	O9—C8—C10	120.3 (2)
S1—C2—H2	123.5	C11—C10—C8	123.2 (2)
C3—C2—S1	113.0 (2)	C15—C10—C8	119.10 (19)
C3—C2—H2	123.5	C15—C10—C11	117.7 (2)
C2—C3—C4	110.3 (2)	C10—C11—H11	119.4
C2—C3—C6	123.0 (2)	C12—C11—C10	121.3 (2)
C4—C3—C6	126.6 (2)	C12—C11—H11	119.4
C3—C4—H4	123.3	C11—C12—H12	120.1
C5—C4—C3	113.3 (2)	C11—C12—C13	119.8 (2)
C5—C4—H4	123.3	C13—C12—H12	120.1
S1—C5—H5	124.5	C12—C13—C14	119.7 (2)
C4—C5—S1	111.0 (2)	O16—C13—C12	122.6 (2)
C4—C5—H5	124.5	O16—C13—C14	117.8 (2)
C3—C6—H6	116.6	C13—C14—H14	120.0
C7—C6—C3	126.9 (2)	C15—C14—C13	120.0 (2)
C7—C6—H6	116.6	C15—C14—H14	120.0
C6—C7—H7	119.0	C10—C15—H15	119.3
C6—C7—C8	122.0 (2)	C14—C15—C10	121.4 (2)
C8—C7—H7	119.0	C14—C15—H15	119.3
C7—C8—C10	119.85 (19)	C13—O16—H16	109.5
O9—C8—C7	119.9 (2)		
			
S1—C2—C3—C4	0.1 (3)	C7—C8—C10—C15	167.8 (2)
S1—C2—C3—C6	−177.0 (2)	C8—C10—C11—C12	−176.7 (2)
C2—S1—C5—C4	0.5 (3)	C8—C10—C15—C14	178.6 (2)
C2—C3—C4—C5	0.3 (4)	O9—C8—C10—C11	165.4 (2)
C2—C3—C6—C7	179.4 (3)	O9—C8—C10—C15	−13.6 (3)
C3—C4—C5—S1	−0.5 (3)	C10—C11—C12—C13	−2.3 (4)
C3—C6—C7—C8	−175.4 (2)	C11—C10—C15—C14	−0.5 (3)
C4—C3—C6—C7	2.7 (4)	C11—C12—C13—C14	0.3 (3)
C5—S1—C2—C3	−0.3 (3)	C11—C12—C13—O16	−179.7 (2)
C6—C3—C4—C5	177.3 (2)	C12—C13—C14—C15	1.6 (3)
C6—C7—C8—O9	9.1 (4)	C13—C14—C15—C10	−1.5 (4)
C6—C7—C8—C10	−172.3 (2)	C15—C10—C11—C12	2.4 (3)
C7—C8—C10—C11	−13.2 (3)	O16—C13—C14—C15	−178.5 (2)

### 1-(4-Hydroxyphenyl)-3-(thiophen-3-yl)prop-1-en-3-one (1) Hydrogen-bond geometry (Å, º)

**Table:**

*D*—H···*A*	*D*—H	H···*A*	*D*···*A*	*D*—H···*A*
O16—H16···O9^i^	0.82	1.86	2.667 (2)	167
C6—H6···O9	0.93	2.46	2.785 (3)	100
C11—H11···O16^ii^	0.93	2.55	3.425 (3)	157

Symmetry codes: (i) *x*+1/2, *y*, −*z*+3/2; (ii) −*x*+2, *y*−1/2, −*z*+3/2.

### 1-(4-Methoxyphenyl)-3-(thiophen-3-yl)prop-1-en-3-one (2) Crystal data

**Table:**

C_14_H_12_O_2_S	*F*(000) = 512
*M**_r_* = 244.30	*D*_x_ = 1.350 Mg m^−^^3^
Monoclinic, *P*2_1_/*c*	Mo *K*α radiation, λ = 0.71073 Å
*a* = 16.4118 (13) Å	Cell parameters from 1920 reflections
*b* = 5.8387 (5) Å	θ = 3.2–27.3°
*c* = 12.6456 (9) Å	µ = 0.26 mm^−^^1^
β = 97.279 (7)°	*T* = 294 K
*V* = 1201.98 (16) Å^3^	Block, white
*Z* = 4	0.45 × 0.3 × 0.15 mm

### 1-(4-Methoxyphenyl)-3-(thiophen-3-yl)prop-1-en-3-one (2) Data collection

**Table:**

SuperNova, single source at offset/far, Eos diffractometer	2457 independent reflections
Radiation source: micro-focus sealed X-ray tube, SuperNova (Mo) X-ray Source	1771 reflections with *I* > 2σ(*I*)
Mirror monochromator	*R*_int_ = 0.021
Detector resolution: 15.9631 pixels mm^-1^	θ_max_ = 26.4°, θ_min_ = 2.5°
ω scans	*h* = −19→20
Absorption correction: multi-scan (CrysAlisPro; Rigaku OD, 2018)	*k* = −4→7
*T*_min_ = 0.803, *T*_max_ = 1.000	*l* = −15→15
5075 measured reflections	

### 1-(4-Methoxyphenyl)-3-(thiophen-3-yl)prop-1-en-3-one (2) Refinement

**Table:**

Refinement on *F*^2^	Hydrogen site location: inferred from neighbouring sites
Least-squares matrix: full	H-atom parameters constrained
*R*[*F*^2^ > 2σ(*F*^2^)] = 0.046	*w* = 1/[σ^2^(*F*_o_^2^) + (0.0416*P*)^2^ + 0.3903*P*] where *P* = (*F*_o_^2^ + 2*F*_c_^2^)/3
*wR*(*F*^2^) = 0.118	(Δ/σ)_max_ < 0.001
*S* = 1.04	Δρ_max_ = 0.16 e Å^−^^3^
2457 reflections	Δρ_min_ = −0.26 e Å^−^^3^
156 parameters	Extinction correction: SHELXL-2016/4 (Sheldrick 2015), Fc^*^=kFc[1+0.001xFc^2^λ^3^/sin(2θ)]^-1/4^
0 restraints	Extinction coefficient: 0.0141 (16)

### 1-(4-Methoxyphenyl)-3-(thiophen-3-yl)prop-1-en-3-one (2) Special details

**Table:**

Geometry. All esds (except the esd in the dihedral angle between two l.s. planes) are estimated using the full covariance matrix. The cell esds are taken into account individually in the estimation of esds in distances, angles and torsion angles; correlations between esds in cell parameters are only used when they are defined by crystal symmetry. An approximate (isotropic) treatment of cell esds is used for estimating esds involving l.s. planes.

### 1-(4-Methoxyphenyl)-3-(thiophen-3-yl)prop-1-en-3-one (2) Fractional atomic coordinates and isotropic or equivalent isotropic displacement parameters (Å^2^)

**Table:**

	*x*	*y*	*z*	*U*_iso_*/*U*_eq_	
S1	0.04793 (4)	0.76040 (12)	0.07886 (5)	0.0654 (3)	
C2	0.10560 (14)	0.9740 (4)	0.14100 (16)	0.0519 (6)	
H2	0.116506	1.111424	0.108336	0.062*	
C3	0.13426 (12)	0.9216 (4)	0.24447 (15)	0.0421 (5)	
C4	0.10869 (13)	0.6985 (4)	0.27105 (18)	0.0494 (6)	
H4	0.122905	0.631591	0.337572	0.059*	
C5	0.06133 (14)	0.5929 (4)	0.18926 (18)	0.0558 (6)	
H5	0.039091	0.447129	0.193370	0.067*	
C6	0.18106 (12)	1.0798 (4)	0.31699 (16)	0.0463 (5)	
H6	0.191362	1.224036	0.290462	0.056*	
C7	0.21052 (13)	1.0373 (4)	0.41779 (16)	0.0477 (5)	
H7	0.202752	0.892707	0.445659	0.057*	
C8	0.25502 (13)	1.2111 (4)	0.48691 (17)	0.0468 (5)	
O9	0.25416 (11)	1.4134 (3)	0.46020 (13)	0.0657 (5)	
C10	0.30162 (12)	1.1378 (4)	0.58960 (15)	0.0430 (5)	
C11	0.28855 (13)	0.9319 (4)	0.63854 (16)	0.0499 (6)	
H11	0.249747	0.830772	0.605098	0.060*	
C12	0.33136 (13)	0.8718 (4)	0.73559 (16)	0.0522 (6)	
H12	0.320424	0.733792	0.767704	0.063*	
C13	0.39048 (13)	1.0180 (4)	0.78457 (16)	0.0467 (5)	
C14	0.40604 (15)	1.2229 (4)	0.73538 (19)	0.0588 (7)	
H14	0.446764	1.320516	0.767118	0.071*	
C15	0.36170 (15)	1.2817 (4)	0.64040 (18)	0.0565 (6)	
H15	0.371979	1.421195	0.609103	0.068*	
O16	0.43757 (9)	0.9765 (3)	0.87912 (11)	0.0586 (5)	
C17	0.42397 (17)	0.7696 (5)	0.93373 (19)	0.0679 (7)	
H17A	0.367631	0.762376	0.946635	0.102*	
H17B	0.459086	0.765633	1.000460	0.102*	
H17C	0.436021	0.641159	0.890914	0.102*	

### 1-(4-Methoxyphenyl)-3-(thiophen-3-yl)prop-1-en-3-one (2) Atomic displacement parameters (Å^2^)

**Table:**

	*U*^11^	*U*^22^	*U*^33^	*U*^12^	*U*^13^	*U*^23^
S1	0.0659 (4)	0.0704 (5)	0.0552 (4)	0.0009 (3)	−0.0104 (3)	−0.0052 (3)
C2	0.0571 (13)	0.0487 (14)	0.0489 (12)	0.0044 (11)	0.0019 (10)	0.0049 (11)
C3	0.0414 (11)	0.0426 (13)	0.0418 (11)	0.0067 (9)	0.0036 (9)	0.0015 (10)
C4	0.0497 (12)	0.0462 (14)	0.0515 (12)	0.0020 (10)	0.0033 (10)	0.0054 (11)
C5	0.0510 (13)	0.0420 (13)	0.0730 (15)	−0.0027 (11)	0.0029 (11)	−0.0003 (12)
C6	0.0475 (12)	0.0419 (13)	0.0489 (12)	0.0019 (10)	0.0042 (10)	0.0038 (10)
C7	0.0507 (12)	0.0444 (13)	0.0470 (12)	−0.0033 (10)	0.0027 (10)	0.0024 (11)
C8	0.0487 (12)	0.0454 (14)	0.0469 (12)	−0.0009 (10)	0.0086 (9)	0.0006 (11)
O9	0.0854 (13)	0.0450 (10)	0.0631 (10)	−0.0029 (9)	−0.0039 (9)	0.0039 (9)
C10	0.0458 (11)	0.0421 (12)	0.0415 (11)	−0.0038 (10)	0.0072 (9)	−0.0039 (10)
C11	0.0523 (13)	0.0500 (14)	0.0455 (12)	−0.0152 (11)	−0.0013 (10)	−0.0011 (11)
C12	0.0595 (14)	0.0490 (14)	0.0465 (12)	−0.0131 (11)	0.0007 (10)	0.0038 (11)
C13	0.0449 (12)	0.0523 (14)	0.0420 (11)	−0.0022 (10)	0.0026 (9)	−0.0064 (11)
C14	0.0611 (14)	0.0541 (15)	0.0578 (14)	−0.0200 (12)	−0.0058 (11)	−0.0068 (12)
C15	0.0686 (15)	0.0452 (14)	0.0544 (13)	−0.0158 (12)	0.0027 (11)	0.0005 (11)
O16	0.0583 (10)	0.0640 (11)	0.0495 (9)	−0.0088 (8)	−0.0083 (7)	−0.0020 (8)
C17	0.0807 (18)	0.0666 (18)	0.0513 (14)	−0.0019 (14)	−0.0108 (12)	0.0017 (13)

### 1-(4-Methoxyphenyl)-3-(thiophen-3-yl)prop-1-en-3-one (2) Geometric parameters (Å, º)

**Table:**

S1—C2	1.697 (2)	C10—C11	1.381 (3)
S1—C5	1.696 (2)	C10—C15	1.390 (3)
C2—H2	0.9300	C11—H11	0.9300
C2—C3	1.368 (3)	C11—C12	1.380 (3)
C3—C4	1.422 (3)	C12—H12	0.9300
C3—C6	1.451 (3)	C12—C13	1.379 (3)
C4—H4	0.9300	C13—C14	1.387 (3)
C4—C5	1.360 (3)	C13—O16	1.361 (2)
C5—H5	0.9300	C14—H14	0.9300
C6—H6	0.9300	C14—C15	1.367 (3)
C6—C7	1.328 (3)	C15—H15	0.9300
C7—H7	0.9300	O16—C17	1.423 (3)
C7—C8	1.471 (3)	C17—H17A	0.9600
C8—O9	1.228 (3)	C17—H17B	0.9600
C8—C10	1.484 (3)	C17—H17C	0.9600
			
C5—S1—C2	92.07 (11)	C15—C10—C8	119.1 (2)
S1—C2—H2	123.8	C10—C11—H11	119.0
C3—C2—S1	112.49 (18)	C12—C11—C10	122.0 (2)
C3—C2—H2	123.8	C12—C11—H11	119.0
C2—C3—C4	110.8 (2)	C11—C12—H12	120.3
C2—C3—C6	123.5 (2)	C13—C12—C11	119.5 (2)
C4—C3—C6	125.60 (19)	C13—C12—H12	120.3
C3—C4—H4	123.4	C12—C13—C14	119.44 (19)
C5—C4—C3	113.1 (2)	O16—C13—C12	125.0 (2)
C5—C4—H4	123.4	O16—C13—C14	115.54 (19)
S1—C5—H5	124.3	C13—C14—H14	119.9
C4—C5—S1	111.50 (18)	C15—C14—C13	120.2 (2)
C4—C5—H5	124.3	C15—C14—H14	119.9
C3—C6—H6	117.0	C10—C15—H15	119.3
C7—C6—C3	125.9 (2)	C14—C15—C10	121.4 (2)
C7—C6—H6	117.0	C14—C15—H15	119.3
C6—C7—H7	118.8	C13—O16—C17	118.08 (18)
C6—C7—C8	122.3 (2)	O16—C17—H17A	109.5
C8—C7—H7	118.8	O16—C17—H17B	109.5
C7—C8—C10	118.9 (2)	O16—C17—H17C	109.5
O9—C8—C7	120.8 (2)	H17A—C17—H17B	109.5
O9—C8—C10	120.3 (2)	H17A—C17—H17C	109.5
C11—C10—C8	123.42 (19)	H17B—C17—H17C	109.5
C11—C10—C15	117.43 (19)		
			
S1—C2—C3—C4	1.0 (2)	C8—C10—C11—C12	−178.1 (2)
S1—C2—C3—C6	−176.32 (16)	C8—C10—C15—C14	179.6 (2)
C2—S1—C5—C4	−0.02 (19)	O9—C8—C10—C11	162.0 (2)
C2—C3—C4—C5	−1.1 (3)	O9—C8—C10—C15	−17.9 (3)
C2—C3—C6—C7	−179.7 (2)	C10—C11—C12—C13	−1.6 (4)
C3—C4—C5—S1	0.6 (3)	C11—C10—C15—C14	−0.3 (4)
C3—C6—C7—C8	−177.8 (2)	C11—C12—C13—C14	−0.1 (3)
C4—C3—C6—C7	3.3 (4)	C11—C12—C13—O16	−178.9 (2)
C5—S1—C2—C3	−0.60 (18)	C12—C13—C14—C15	1.6 (4)
C6—C3—C4—C5	176.2 (2)	C12—C13—O16—C17	−1.6 (3)
C6—C7—C8—O9	13.0 (3)	C13—C14—C15—C10	−1.4 (4)
C6—C7—C8—C10	−166.6 (2)	C14—C13—O16—C17	179.5 (2)
C7—C8—C10—C11	−18.4 (3)	C15—C10—C11—C12	1.8 (3)
C7—C8—C10—C15	161.7 (2)	O16—C13—C14—C15	−179.5 (2)

### 1-(4-Methoxyphenyl)-3-(thiophen-3-yl)prop-1-en-3-one (2) Hydrogen-bond geometry (Å, º)

*Cg*1 is the centroid of the S1/C2–C5 ring.

**Table:**

*D*—H···*A*	*D*—H	H···*A*	*D*···*A*	*D*—H···*A*
C5—H5···*Cg*1^i^	0.93	2.94	3.602 (2)	129
C11—H11···*Cg*1^ii^	0.93	2.99	3.598 (2)	125

Symmetry codes: (i) −*x*, *y*−1/2, −*z*+1/2; (ii) *x*, −*y*+3/2, *z*+1/2.

### 1-(4-Ethoxyphenyl)-3-(thiophen-3-yl)prop-1-en-3-one (3) Crystal data

**Table:**

C_15_H_14_O_2_S	*F*(000) = 544
*M**_r_* = 258.32	*D*_x_ = 1.281 Mg m^−^^3^
Monoclinic, *P*2_1_/*c*	Mo *K*α radiation, λ = 0.71073 Å
*a* = 16.5120 (8) Å	Cell parameters from 4827 reflections
*b* = 7.7851 (5) Å	θ = 3.3–27.9°
*c* = 10.4913 (5) Å	µ = 0.23 mm^−^^1^
β = 96.813 (4)°	*T* = 294 K
*V* = 1339.11 (13) Å^3^	Block, white
*Z* = 4	0.5 × 0.35 × 0.15 mm

### 1-(4-Ethoxyphenyl)-3-(thiophen-3-yl)prop-1-en-3-one (3) Data collection

**Table:**

SuperNova, single source at offset/far, Eos diffractometer	2734 independent reflections
Radiation source: micro-focus sealed X-ray tube, SuperNova (Mo) X-ray Source	2162 reflections with *I* > 2σ(*I*)
Mirror monochromator	*R*_int_ = 0.035
Detector resolution: 15.9631 pixels mm^-1^	θ_max_ = 26.4°, θ_min_ = 2.5°
ω scans	*h* = −20→20
Absorption correction: multi-scan (CrysAlisPro; Rigaku OD, 2018)	*k* = −9→9
*T*_min_ = 0.733, *T*_max_ = 1.000	*l* = −12→13
13246 measured reflections	

### 1-(4-Ethoxyphenyl)-3-(thiophen-3-yl)prop-1-en-3-one (3) Refinement

**Table:**

Refinement on *F*^2^	Hydrogen site location: inferred from neighbouring sites
Least-squares matrix: full	H-atom parameters constrained
*R*[*F*^2^ > 2σ(*F*^2^)] = 0.047	*w* = 1/[σ^2^(*F*_o_^2^) + (0.0633*P*)^2^ + 0.4069*P*] where *P* = (*F*_o_^2^ + 2*F*_c_^2^)/3
*wR*(*F*^2^) = 0.141	(Δ/σ)_max_ < 0.001
*S* = 1.05	Δρ_max_ = 0.19 e Å^−^^3^
2734 reflections	Δρ_min_ = −0.28 e Å^−^^3^
165 parameters	Extinction correction: SHELXL-2016/4 (Sheldrick 2015), Fc^*^=kFc[1+0.001xFc^2^λ^3^/sin(2θ)]^-1/4^
0 restraints	Extinction coefficient: 0.0073 (17)

### 1-(4-Ethoxyphenyl)-3-(thiophen-3-yl)prop-1-en-3-one (3) Special details

**Table:**

Geometry. All esds (except the esd in the dihedral angle between two l.s. planes) are estimated using the full covariance matrix. The cell esds are taken into account individually in the estimation of esds in distances, angles and torsion angles; correlations between esds in cell parameters are only used when they are defined by crystal symmetry. An approximate (isotropic) treatment of cell esds is used for estimating esds involving l.s. planes.

### 1-(4-Ethoxyphenyl)-3-(thiophen-3-yl)prop-1-en-3-one (3) Fractional atomic coordinates and isotropic or equivalent isotropic displacement parameters (Å^2^)

**Table:**

	*x*	*y*	*z*	*U*_iso_*/*U*_eq_	
S1	0.28921 (3)	0.41932 (9)	0.57261 (6)	0.0697 (3)	
C2	0.35583 (12)	0.4427 (3)	0.70816 (19)	0.0558 (5)	
H2	0.340852	0.481608	0.785855	0.067*	
C3	0.43322 (11)	0.3985 (2)	0.69052 (17)	0.0458 (4)	
C4	0.43736 (12)	0.3435 (3)	0.56166 (18)	0.0551 (5)	
H4	0.485324	0.307932	0.531318	0.066*	
C5	0.36332 (13)	0.3486 (3)	0.4872 (2)	0.0588 (5)	
H5	0.354921	0.317238	0.401153	0.071*	
C6	0.50131 (12)	0.4082 (2)	0.79107 (18)	0.0483 (5)	
H6	0.489859	0.443268	0.871763	0.058*	
C7	0.57803 (12)	0.3718 (3)	0.77867 (18)	0.0518 (5)	
H7	0.591201	0.335993	0.699110	0.062*	
C8	0.64340 (12)	0.3860 (3)	0.88615 (17)	0.0490 (5)	
O9	0.62809 (9)	0.3934 (2)	0.99739 (13)	0.0670 (5)	
C10	0.72986 (12)	0.3954 (2)	0.85742 (17)	0.0476 (5)	
C11	0.75356 (12)	0.3597 (3)	0.73833 (18)	0.0540 (5)	
H11	0.714251	0.329023	0.671266	0.065*	
C12	0.83455 (13)	0.3684 (3)	0.7166 (2)	0.0594 (5)	
H12	0.849281	0.344313	0.635592	0.071*	
C13	0.89322 (13)	0.4129 (3)	0.8157 (2)	0.0568 (5)	
C14	0.87043 (13)	0.4512 (3)	0.9360 (2)	0.0618 (6)	
H14	0.909675	0.483056	1.002823	0.074*	
C15	0.79065 (13)	0.4420 (3)	0.95555 (19)	0.0571 (5)	
H15	0.776104	0.467265	1.036427	0.069*	
O16	0.97471 (9)	0.4227 (2)	0.80525 (16)	0.0762 (5)	
C17	1.00141 (16)	0.3853 (4)	0.6849 (3)	0.0901 (9)	
H17A	0.978364	0.467414	0.621098	0.108*	
H17B	0.983604	0.271209	0.656990	0.108*	
C18	1.09277 (17)	0.3952 (5)	0.6991 (4)	0.1138 (13)	
H18A	1.111988	0.364258	0.619307	0.171*	
H18B	1.115025	0.317438	0.765128	0.171*	
H18C	1.109771	0.510223	0.721773	0.171*	

### 1-(4-Ethoxyphenyl)-3-(thiophen-3-yl)prop-1-en-3-one (3) Atomic displacement parameters (Å^2^)

**Table:**

	*U*^11^	*U*^22^	*U*^33^	*U*^12^	*U*^13^	*U*^23^
S1	0.0502 (4)	0.0898 (5)	0.0684 (4)	0.0040 (3)	0.0052 (3)	0.0000 (3)
C2	0.0549 (12)	0.0668 (13)	0.0479 (11)	0.0079 (10)	0.0153 (9)	0.0016 (9)
C3	0.0481 (10)	0.0477 (10)	0.0430 (10)	0.0015 (8)	0.0115 (8)	0.0017 (8)
C4	0.0505 (11)	0.0663 (13)	0.0500 (11)	0.0015 (9)	0.0128 (9)	−0.0087 (9)
C5	0.0619 (13)	0.0676 (13)	0.0472 (11)	0.0005 (10)	0.0072 (9)	−0.0116 (10)
C6	0.0522 (11)	0.0544 (11)	0.0397 (9)	0.0043 (8)	0.0118 (8)	0.0013 (8)
C7	0.0507 (11)	0.0654 (13)	0.0400 (10)	0.0021 (9)	0.0090 (8)	−0.0020 (9)
C8	0.0506 (11)	0.0582 (12)	0.0387 (10)	0.0054 (9)	0.0077 (8)	0.0025 (8)
O9	0.0584 (9)	0.1032 (13)	0.0407 (8)	0.0094 (8)	0.0111 (6)	0.0024 (7)
C10	0.0506 (11)	0.0527 (11)	0.0393 (9)	0.0030 (8)	0.0047 (8)	0.0035 (8)
C11	0.0491 (11)	0.0707 (14)	0.0422 (10)	−0.0022 (9)	0.0049 (8)	−0.0041 (9)
C12	0.0534 (12)	0.0780 (15)	0.0476 (11)	−0.0027 (10)	0.0098 (9)	−0.0075 (10)
C13	0.0461 (11)	0.0669 (14)	0.0580 (12)	−0.0025 (9)	0.0089 (9)	0.0003 (10)
C14	0.0550 (12)	0.0813 (16)	0.0472 (11)	−0.0048 (11)	−0.0020 (9)	−0.0035 (10)
C15	0.0553 (12)	0.0759 (14)	0.0398 (10)	0.0003 (10)	0.0045 (9)	−0.0005 (9)
O16	0.0476 (9)	0.1096 (15)	0.0726 (11)	−0.0107 (8)	0.0115 (7)	−0.0139 (9)
C17	0.0590 (15)	0.121 (2)	0.095 (2)	−0.0131 (14)	0.0279 (14)	−0.0264 (17)
C18	0.0593 (16)	0.135 (3)	0.153 (3)	−0.0193 (16)	0.0391 (18)	−0.049 (2)

### 1-(4-Ethoxyphenyl)-3-(thiophen-3-yl)prop-1-en-3-one (3) Geometric parameters (Å, º)

**Table:**

S1—C2	1.701 (2)	C11—H11	0.9300
S1—C5	1.692 (2)	C11—C12	1.385 (3)
C2—H2	0.9300	C12—H12	0.9300
C2—C3	1.357 (3)	C12—C13	1.379 (3)
C3—C4	1.427 (3)	C13—C14	1.392 (3)
C3—C6	1.450 (3)	C13—O16	1.365 (3)
C4—H4	0.9300	C14—H14	0.9300
C4—C5	1.371 (3)	C14—C15	1.359 (3)
C5—H5	0.9300	C15—H15	0.9300
C6—H6	0.9300	O16—C17	1.416 (3)
C6—C7	1.319 (3)	C17—H17A	0.9700
C7—H7	0.9300	C17—H17B	0.9700
C7—C8	1.470 (3)	C17—C18	1.500 (4)
C8—O9	1.224 (2)	C18—H18A	0.9600
C8—C10	1.496 (3)	C18—H18B	0.9600
C10—C11	1.381 (3)	C18—H18C	0.9600
C10—C15	1.398 (3)		
			
C5—S1—C2	92.34 (10)	C12—C11—H11	119.3
S1—C2—H2	123.7	C11—C12—H12	120.2
C3—C2—S1	112.62 (15)	C13—C12—C11	119.60 (19)
C3—C2—H2	123.7	C13—C12—H12	120.2
C2—C3—C4	110.92 (18)	C12—C13—C14	119.8 (2)
C2—C3—C6	123.30 (17)	O16—C13—C12	124.39 (19)
C4—C3—C6	125.79 (17)	O16—C13—C14	115.84 (19)
C3—C4—H4	123.5	C13—C14—H14	120.1
C5—C4—C3	113.01 (18)	C15—C14—C13	119.78 (19)
C5—C4—H4	123.5	C15—C14—H14	120.1
S1—C5—H5	124.4	C10—C15—H15	119.1
C4—C5—S1	111.11 (15)	C14—C15—C10	121.80 (19)
C4—C5—H5	124.4	C14—C15—H15	119.1
C3—C6—H6	117.0	C13—O16—C17	118.30 (18)
C7—C6—C3	126.00 (18)	O16—C17—H17A	110.0
C7—C6—H6	117.0	O16—C17—H17B	110.0
C6—C7—H7	118.8	O16—C17—C18	108.5 (2)
C6—C7—C8	122.31 (18)	H17A—C17—H17B	108.4
C8—C7—H7	118.8	C18—C17—H17A	110.0
C7—C8—C10	118.74 (16)	C18—C17—H17B	110.0
O9—C8—C7	121.26 (18)	C17—C18—H18A	109.5
O9—C8—C10	119.99 (17)	C17—C18—H18B	109.5
C11—C10—C8	123.51 (17)	C17—C18—H18C	109.5
C11—C10—C15	117.56 (18)	H18A—C18—H18B	109.5
C15—C10—C8	118.92 (17)	H18A—C18—H18C	109.5
C10—C11—H11	119.3	H18B—C18—H18C	109.5
C10—C11—C12	121.47 (19)		
			
S1—C2—C3—C4	−0.1 (2)	C8—C10—C15—C14	179.3 (2)
S1—C2—C3—C6	179.55 (15)	O9—C8—C10—C11	168.8 (2)
C2—S1—C5—C4	−0.05 (18)	O9—C8—C10—C15	−10.9 (3)
C2—C3—C4—C5	0.1 (3)	C10—C11—C12—C13	0.3 (3)
C2—C3—C6—C7	−177.9 (2)	C11—C10—C15—C14	−0.4 (3)
C3—C4—C5—S1	0.0 (2)	C11—C12—C13—C14	−1.0 (3)
C3—C6—C7—C8	179.75 (18)	C11—C12—C13—O16	178.9 (2)
C4—C3—C6—C7	1.8 (3)	C12—C13—C14—C15	1.0 (3)
C5—S1—C2—C3	0.11 (18)	C12—C13—O16—C17	0.2 (3)
C6—C3—C4—C5	−179.58 (19)	C13—C14—C15—C10	−0.3 (4)
C6—C7—C8—O9	17.1 (3)	C13—O16—C17—C18	−177.0 (2)
C6—C7—C8—C10	−161.57 (19)	C14—C13—O16—C17	−179.9 (2)
C7—C8—C10—C11	−12.6 (3)	C15—C10—C11—C12	0.4 (3)
C7—C8—C10—C15	167.75 (19)	O16—C13—C14—C15	−178.9 (2)
C8—C10—C11—C12	−179.3 (2)		

### 1-(4-Ethoxyphenyl)-3-(thiophen-3-yl)prop-1-en-3-one (3) Hydrogen-bond geometry (Å, º)

**Table:**

*D*—H···*A*	*D*—H	H···*A*	*D*···*A*	*D*—H···*A*
C2—H2···O9^i^	0.93	2.47	3.324 (2)	153

Symmetry code: (i) −*x*+1, −*y*+1, −*z*+2.

### 1-(4-Bromophenyl)-3-(thiophen-3-yl)prop-1-en-3-one (4) Crystal data

**Table:**

C_13_H_9_BrOS	*F*(000) = 584
*M**_r_* = 293.17	*D*_x_ = 1.667 Mg m^−^^3^
Monoclinic, *P*2_1_/*c*	Mo *K*α radiation, λ = 0.71073 Å
*a* = 14.1245 (7) Å	Cell parameters from 4399 reflections
*b* = 14.2016 (13) Å	θ = 2.9–27.3°
*c* = 5.8809 (4) Å	µ = 3.67 mm^−^^1^
β = 98.081 (6)°	*T* = 293 K
*V* = 1167.93 (15) Å^3^	Plate, yellow
*Z* = 4	0.4 × 0.4 × 0.05 mm

### 1-(4-Bromophenyl)-3-(thiophen-3-yl)prop-1-en-3-one (4) Data collection

**Table:**

SuperNova, single source at offset/far, Eos diffractometer	2392 independent reflections
Radiation source: micro-focus sealed X-ray tube, SuperNova (Mo) X-ray Source	1683 reflections with *I* > 2σ(*I*)
Mirror monochromator	*R*_int_ = 0.045
Detector resolution: 15.9631 pixels mm^-1^	θ_max_ = 26.4°, θ_min_ = 2.9°
ω scans	*h* = −17→17
Absorption correction: multi-scan (CrysAlisPro; Rigaku OD, 2018)	*k* = −17→17
*T*_min_ = 0.367, *T*_max_ = 1.000	*l* = −7→7
12050 measured reflections	

### 1-(4-Bromophenyl)-3-(thiophen-3-yl)prop-1-en-3-one (4) Refinement

**Table:**

Refinement on *F*^2^	20 restraints
Least-squares matrix: full	Hydrogen site location: inferred from neighbouring sites
*R*[*F*^2^ > 2σ(*F*^2^)] = 0.043	H-atom parameters constrained
*wR*(*F*^2^) = 0.107	*w* = 1/[σ^2^(*F*_o_^2^) + (0.0407*P*)^2^ + 0.7623*P*] where *P* = (*F*_o_^2^ + 2*F*_c_^2^)/3
*S* = 1.02	(Δ/σ)_max_ = 0.001
2392 reflections	Δρ_max_ = 0.40 e Å^−^^3^
158 parameters	Δρ_min_ = −0.46 e Å^−^^3^

### 1-(4-Bromophenyl)-3-(thiophen-3-yl)prop-1-en-3-one (4) Special details

**Table:**

Geometry. All esds (except the esd in the dihedral angle between two l.s. planes) are estimated using the full covariance matrix. The cell esds are taken into account individually in the estimation of esds in distances, angles and torsion angles; correlations between esds in cell parameters are only used when they are defined by crystal symmetry. An approximate (isotropic) treatment of cell esds is used for estimating esds involving l.s. planes.

### 1-(4-Bromophenyl)-3-(thiophen-3-yl)prop-1-en-3-one (4) Fractional atomic coordinates and isotropic or equivalent isotropic displacement parameters (Å^2^)

**Table:**

	*x*	*y*	*z*	*U*_iso_*/*U*_eq_	Occ. (<1)
S1A	0.07255 (13)	0.3898 (2)	0.4329 (4)	0.0574 (6)	0.702 (4)
S1B	0.1418 (5)	0.3457 (7)	0.2749 (13)	0.0574 (6)	0.298 (4)
C2A	0.1532 (7)	0.412 (2)	0.663 (3)	0.048 (3)	0.702 (4)
H2A	0.137355	0.439712	0.795694	0.058*	0.702 (4)
C2B	0.2476 (16)	0.341 (5)	0.438 (6)	0.048 (3)	0.298 (4)
H2B	0.301123	0.310516	0.397088	0.058*	0.298 (4)
C3	0.2452 (2)	0.3858 (2)	0.6388 (5)	0.0417 (8)	
C4A	0.2461 (7)	0.3456 (17)	0.416 (2)	0.048 (3)	0.702 (4)
H4A	0.302496	0.326195	0.365401	0.058*	0.702 (4)
C4B	0.1551 (18)	0.422 (5)	0.664 (6)	0.048 (3)	0.298 (4)
H4B	0.142572	0.455562	0.792513	0.058*	0.298 (4)
C5A	0.1586 (7)	0.3375 (9)	0.2809 (18)	0.053 (3)	0.702 (4)
H5A	0.146845	0.309732	0.136500	0.063*	0.702 (4)
C5B	0.0855 (10)	0.4021 (19)	0.475 (3)	0.053 (3)	0.298 (4)
H5B	0.020804	0.416891	0.461047	0.063*	0.298 (4)
C6	0.3264 (2)	0.3933 (2)	0.8186 (6)	0.0451 (8)	
H6	0.313682	0.414696	0.960622	0.054*	
C7	0.4168 (2)	0.3729 (2)	0.8023 (6)	0.0485 (9)	
H7	0.433284	0.356673	0.659823	0.058*	
C8	0.4916 (3)	0.3752 (2)	1.0027 (6)	0.0460 (8)	
O9	0.4718 (2)	0.3764 (2)	1.1988 (4)	0.0676 (8)	
C10	0.5938 (2)	0.3754 (2)	0.9658 (5)	0.0403 (8)	
C11	0.6238 (2)	0.4075 (2)	0.7646 (6)	0.0451 (8)	
H11	0.578794	0.428136	0.644229	0.054*	
C12	0.7202 (3)	0.4092 (3)	0.7410 (6)	0.0470 (8)	
H12	0.740087	0.432427	0.607649	0.056*	
C13	0.7860 (2)	0.3760 (2)	0.9180 (6)	0.0434 (8)	
C14	0.7584 (3)	0.3438 (3)	1.1203 (6)	0.0491 (9)	
H14	0.803570	0.322082	1.239069	0.059*	
C15	0.6624 (2)	0.3447 (2)	1.1429 (6)	0.0447 (8)	
H15	0.643292	0.324227	1.279603	0.054*	
Br16	0.91660 (3)	0.37156 (4)	0.88024 (8)	0.0730 (2)	

### 1-(4-Bromophenyl)-3-(thiophen-3-yl)prop-1-en-3-one (4) Atomic displacement parameters (Å^2^)

**Table:**

	*U*^11^	*U*^22^	*U*^33^	*U*^12^	*U*^13^	*U*^23^
S1A	0.0459 (8)	0.0718 (14)	0.0532 (11)	0.0051 (8)	0.0029 (6)	−0.0014 (9)
S1B	0.0459 (8)	0.0718 (14)	0.0532 (11)	0.0051 (8)	0.0029 (6)	−0.0014 (9)
C2A	0.044 (4)	0.056 (10)	0.045 (4)	0.006 (4)	0.010 (3)	−0.004 (4)
C2B	0.044 (4)	0.056 (10)	0.045 (4)	0.006 (4)	0.010 (3)	−0.004 (4)
C3	0.0453 (19)	0.0398 (19)	0.0417 (18)	−0.0020 (15)	0.0115 (15)	0.0021 (15)
C4A	0.052 (4)	0.051 (5)	0.043 (5)	−0.002 (3)	0.013 (3)	−0.006 (5)
C4B	0.052 (4)	0.051 (5)	0.043 (5)	−0.002 (3)	0.013 (3)	−0.006 (5)
C5A	0.058 (6)	0.055 (5)	0.051 (4)	0.015 (4)	0.025 (4)	−0.013 (3)
C5B	0.058 (6)	0.055 (5)	0.051 (4)	0.015 (4)	0.025 (4)	−0.013 (3)
C6	0.045 (2)	0.051 (2)	0.0416 (19)	−0.0044 (16)	0.0107 (15)	−0.0040 (15)
C7	0.047 (2)	0.054 (2)	0.0452 (19)	−0.0018 (17)	0.0105 (16)	−0.0068 (16)
C8	0.0447 (19)	0.050 (2)	0.045 (2)	0.0020 (16)	0.0112 (16)	−0.0036 (16)
O9	0.0531 (16)	0.107 (3)	0.0450 (15)	0.0056 (14)	0.0139 (12)	0.0000 (14)
C10	0.0455 (19)	0.0372 (18)	0.0386 (18)	−0.0011 (15)	0.0075 (15)	−0.0038 (14)
C11	0.049 (2)	0.050 (2)	0.0350 (18)	0.0028 (17)	0.0007 (15)	0.0004 (15)
C12	0.054 (2)	0.051 (2)	0.0366 (18)	−0.0055 (17)	0.0101 (16)	0.0013 (16)
C13	0.0382 (18)	0.045 (2)	0.048 (2)	−0.0061 (15)	0.0075 (15)	−0.0056 (16)
C14	0.051 (2)	0.050 (2)	0.0426 (19)	−0.0004 (17)	−0.0036 (16)	0.0041 (16)
C15	0.050 (2)	0.048 (2)	0.0363 (18)	−0.0041 (17)	0.0062 (15)	0.0021 (15)
Br16	0.0430 (3)	0.0970 (4)	0.0803 (4)	−0.0074 (2)	0.0128 (2)	0.0047 (2)

### 1-(4-Bromophenyl)-3-(thiophen-3-yl)prop-1-en-3-one (4) Geometric parameters (Å, º)

**Table:**

S1A—C2A	1.671 (8)	C6—H6	0.9300
C2A—H2A	0.9300	C6—C7	1.325 (5)
S1B—C2B	1.661 (16)	C7—H7	0.9300
C2B—H2B	0.9300	C7—C8	1.469 (5)
C2A—C3	1.378 (9)	C8—O9	1.224 (4)
C2B—C3	1.347 (16)	C8—C10	1.489 (5)
C4A—H4A	0.9300	C10—C11	1.389 (5)
C4B—H4B	0.9300	C10—C15	1.390 (5)
S1A—C5A	1.770 (8)	C11—H11	0.9300
C4A—C5A	1.378 (11)	C11—C12	1.388 (5)
C5A—H5A	0.9300	C12—H12	0.9300
S1B—C5B	1.708 (15)	C12—C13	1.378 (5)
C4B—C5B	1.407 (16)	C13—C14	1.381 (5)
C5B—H5B	0.9300	C13—Br16	1.890 (3)
C3—C4A	1.431 (8)	C14—H14	0.9300
C3—C4B	1.400 (16)	C14—C15	1.381 (5)
C3—C6	1.451 (5)	C15—H15	0.9300
			
C4A—C5A—S1A	107.3 (6)	C7—C6—C3	127.0 (3)
C4B—C5B—S1B	107.4 (9)	C7—C6—H6	116.5
S1A—C2A—H2A	122.9	C6—C7—H7	119.0
S1B—C2B—H2B	124.6	C6—C7—C8	121.9 (3)
C5A—C4A—C3	116.0 (7)	C8—C7—H7	119.0
C5A—C4A—H4A	122.0	C7—C8—C10	119.1 (3)
C5B—C4B—H4B	123.6	O9—C8—C7	121.5 (3)
C2A—S1A—C5A	92.8 (4)	O9—C8—C10	119.5 (3)
S1A—C5A—H5A	126.3	C11—C10—C8	122.9 (3)
C4A—C5A—H5A	126.3	C11—C10—C15	118.5 (3)
C2B—S1B—C5B	95.2 (7)	C15—C10—C8	118.6 (3)
C4B—C5B—H5B	126.3	C10—C11—H11	119.6
S1B—C5B—H5B	126.3	C12—C11—C10	120.8 (3)
C2A—C3—C4A	109.4 (5)	C12—C11—H11	119.6
C2B—C3—C4B	113.8 (9)	C11—C12—H12	120.5
C4A—C3—C6	126.0 (4)	C13—C12—C11	119.1 (3)
C4B—C3—C6	122.2 (7)	C13—C12—H12	120.5
C2B—C3—C6	124.0 (7)	C12—C13—C14	121.5 (3)
C2A—C3—C6	124.5 (5)	C12—C13—Br16	119.2 (3)
C3—C6—H6	116.5	C14—C13—Br16	119.3 (3)
C3—C2A—S1A	114.3 (5)	C13—C14—H14	120.7
C3—C2B—S1B	110.7 (9)	C15—C14—C13	118.7 (3)
C3—C2A—H2A	122.9	C15—C14—H14	120.7
C3—C2B—H2B	124.6	C10—C15—H15	119.3
C3—C4A—H4A	122.0	C14—C15—C10	121.5 (3)
C3—C4B—H4B	123.6	C14—C15—H15	119.3
C3—C4B—C5B	112.7 (11)		
			
C2A—S1A—C5A—C4A	−3 (2)	C6—C3—C4B—C5B	−176 (3)
C2B—S1B—C5B—C4B	4 (5)	C6—C7—C8—O9	15.6 (6)
C5B—S1B—C2B—C3	−4 (5)	C6—C7—C8—C10	−164.9 (3)
C5A—S1A—C2A—C3	2 (2)	C7—C8—C10—C11	24.4 (5)
S1A—C2A—C3—C4A	0 (2)	C7—C8—C10—C15	−157.6 (3)
S1B—C2B—C3—C4B	2 (5)	C8—C10—C11—C12	177.8 (3)
S1B—C2B—C3—C6	179.7 (18)	C8—C10—C15—C14	−179.4 (3)
S1A—C2A—C3—C6	−176.6 (10)	O9—C8—C10—C11	−156.0 (4)
C2A—C3—C6—C7	−177.0 (17)	O9—C8—C10—C15	22.0 (5)
C2B—C3—C6—C7	13 (4)	C10—C11—C12—C13	1.8 (5)
C4A—C3—C6—C7	6.9 (15)	C11—C10—C15—C14	−1.3 (5)
C4B—C3—C6—C7	−170 (4)	C11—C12—C13—C14	−2.0 (5)
C2A—C3—C4A—C5A	−3 (2)	C11—C12—C13—Br16	176.5 (3)
C2B—C3—C4B—C5B	1 (6)	C12—C13—C14—C15	0.5 (5)
C3—C4B—C5B—S1B	−4 (7)	C13—C14—C15—C10	1.2 (5)
C3—C4A—C5A—S1A	4 (2)	C15—C10—C11—C12	−0.2 (5)
C3—C6—C7—C8	−174.3 (3)	Br16—C13—C14—C15	−177.9 (3)
C6—C3—C4A—C5A	173.8 (12)		

### 1-(4-Bromophenyl)-3-(thiophen-3-yl)prop-1-en-3-one (4) Hydrogen-bond geometry (Å, º)

*Cg*1 and *Cg*2 are the centroids of the major- and minor-disorder components of the thiophene ring, respectively.

**Table:**

*D*—H···*A*	*D*—H	H···*A*	*D*···*A*	*D*—H···*A*
C5*A*—H5*A*···*Cg*1^i^	0.93	2.80	3.493 (14)	132
C5*A*—H5*A*···*Cg*2^i^	0.93	2.85	3.52 (2)	130

Symmetry code: (i) *x*, −*y*+1/2, *z*−1/2.
